# Diterpenoids from *Saliva plebeia* R. Br. and Their Antioxidant and Anti-Inflammatory Activities

**DOI:** 10.3390/molecules200814879

**Published:** 2015-08-14

**Authors:** Bao-Bao Zhang, Bai-Qiu He, Jian-Bo Sun, Biao Zeng, Xiao-Ji Shi, Yong Zhou, Ying Niu, Shi-Qi Nie, Feng Feng, Yan Liang, Fei-Hua Wu

**Affiliations:** 1 School of Traditional Chinese Pharmacy, China Pharmaceutical University, Nanjing 211198, China; E-Mails: song212810@163.com (B.-B.Z.); hbqnjfu@163.com (B.-Q.H.); sjb_sp@163.com (J.-B.S.); zengbiao8839@163.com (B.Z.); xjsforever@sina.com (X.-J.S.); zhouyong941026@163.com (Y.Z.); yingniu13@aliyun.com (Y.N.); nie9993939@163.com (S.-Q.N.); fengsunlight@163.com (F.F.); 2 Nanjing Sanhome Pharmaceutical Co., Ltd., Nanjing 210018, China

**Keywords:** *Salvia plebeia*, diterpenoids, antioxidant activity, anti-inflammatory activity

## Abstract

A new skeleton of diterpenoid, 1,2,3,4,4α,9,10,10α-octahydro-(4α-hydroxyymethyl)-1,1-dimethyl-9-(1-methylethyl)-(2*S*,3*S*,4α*R*,9*R*,10α*S*)-2,3,5,7-phenanthrenetertrol, named plebeianiol A (**1**), along with four known diterpenoids (**2**–**5**), were isolated from *Salvia plebeia* R. Br. Their structures were determined on the basis of spectral analysis. In the bioactivity tests, compounds **1**, **2** and **5** showed 1,1-diphenyl-2-picrylhydrazyl (DPPH) radical scavenging activities with IC_50_ values of 20.0–29.6 µM. In addition, these three compounds had significant inhibitory effects on reactive oxygen species (ROS) production in lipopolysaccharide (LPS)-induced macrophages. Compounds **1**–**3** inhibited nitric oxide (NO) production in LPS-induced macrophages with IC_50_ values of 18.0–23.6 µM. These results showed that compounds **1**, **2** had significant antioxidant and anti-inflammatory activities and might provide basis for the treatment of diseases associated with oxidative lesions and inflammation.

## 1. Introduction

*Salvia plebeia* R. Br. is one of the genera from the *Salvia* family. It is widely distributed in many provinces of China. As a traditional Chinese medicine (TCM), it has been used to treat various diseases, such as urinary tract infection and bronchitis [1]. Modern pharmacological studies have shown that it exhibits anti-inflammatory [2], antioxidant [3], antitumor [4], hepatoprotective [5] and antimicrobial [6] activities. Phytochemical studies of *S. plebeia* R. Br. revealed the presence of flavonoids [7], terpenoids [8] and lignans [9].

In the course of researching bioactive components from *S. plebeia* R. Br., we found that ethyl acetate (EtOAc) fraction of *S. plebeia* R. Br. showed significant inhibitory activity against the production of NO in LPS-induced RAW 264.7 macrophages. According to the guidance of bioactivity, a new skeleton of diterpenoid (**1**) and four known diterpenoids (**2**–**5**) were isolated from the bioactive fractions of EtOAc fraction of *S. plebeia* R. Br. In the present study, we reported the isolation, structure elucidation, as well as antioxidant and anti-inflammatory activities of the isolated compounds *in vitro*.

## 2. Results and Discussion

**Figure 1:**
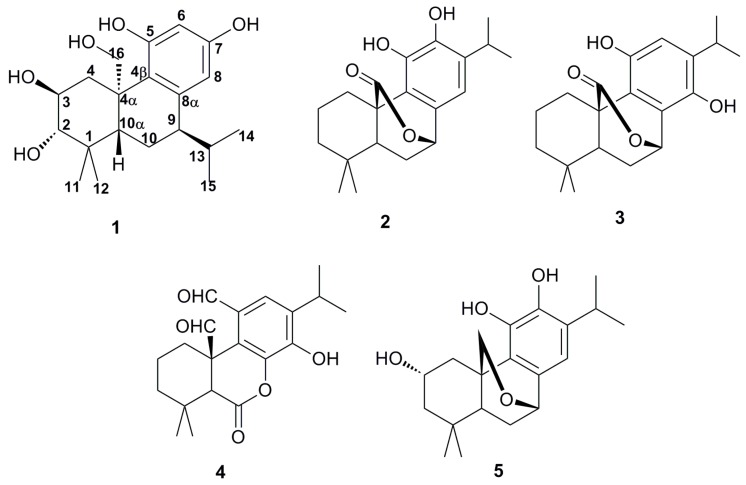
Structures of compounds **1**–**5**.

The 80% aqueous ethanol extract of the air-dried herb of *S. plebeia* R. Br. was successively partitioned with petroleum ether, EtOAc and *N*-butyl alcohol (*n*-BuOH), respectively. Then five diterpenoids (**1**–**5**) (Figure 1) were isolated by successive column of the EtOAc fraction on silica gel, MCI column chromatography and Sephadex LH-20.

Compound **1** was obtained as a yellow crystal. The molecular formula C_20_H_30_O_5_ was determined by high-resolution electrospray ionization mass spectrometry (HR-ESI-MS) (*m*/*z* 351.2159, [M + H]^+^, calcd for 351.2166), indicating six degrees of unsaturation. The melting point (m.p.) was 232–233 °C. The UV spectrum (194 and 211 nm) showed the absorption of conjugated double bond. Its IR spectrum exhibited the presence of hydroxyl (3455 cm^−1^) and phenyl group (1641, 1423, 875 and 699 cm^−1^).

The ^1^H-NMR spectrum showed signals of two independent phenolic hydroxyl groups at δ_H_ 9.61 (1H, brs, OH-5) and δ_H_ 7.54 (1H, brs, OH-7), two independent aromatic protons at δ_H_ 6.53 (1H, brs, H-6) and δ_H_ 6.35 (1H, brs, H-8), two alcohol hydroxyl groups at δ_H_ 4.51 (1H, d, *J* = 3.78 Hz), δ_H_ 4.39 (1H, d, *J* = 4.05 Hz) The ^13^C-NMR spectrum contained six carbon signals on the benzene ring, including δ_C_ 130.2 (C-4β), δ_C_ 143.0 (C-5), δ_C_ 126.3 (C-6), δ_C_ 141.6 (C-7), δ_C_ 117.5 (C-8), δ_C_ 131.9 (C-8α), three carbon signals were correlated with the hydroxyl at δ_C_ 81.8 (C-2), δ_C_ 66.8 (C-3) and δ_C_ 65.3 (C-16) (Table 1).

**Table 1:** ^1^H- (300 MHz) and ^13^C- (75 MHz) NMR data of compound 1 in DMSO-*d*_6_ (TMS, δ ppm; *J* in Hz).

Position	δ_C_	δ_H_	Position	δ_C_	δ_H_
1	38.6		10	18.1	I 1.50 (1H, m); II 1.60 (1H, m)
2	81.8	2.84 (1H, dd, *J* = 3.42, 5.79 Hz)	10α	52.1	1.30 (1H, m)
3	66.8	3.57 (1H, m)	11	29.6	1.01 (3H, s)
4	38.0	I 0.96 (1H, m); II 3.36 (1H, d, *J* = 3.39 Hz)	12	18.1	0.78 (3H, s)
4α	43.8		13	31.7	2.74 (1H, m)
4β	130.2		14	22.3	1.10 (3H, d, *J* = 2.40 Hz)
5	143.0		15	22.4	1.11 (3H, d, *J* = 2.49 Hz)
6	126.3	6.53 (1H, brs)	16	65.3	I 3.73 (1H, d, *J* = 9.42 Hz); II 4.12 (1H, d, *J* = 9.84 Hz)
7	141.6		2-OH		4.51 (1H, d, *J* = 3.78 Hz)
8α	117.5	6.35 (1H, brs)	3-OH		4.39 (1H, d, *J* = 4.05 Hz)
8	131.9		5-OH		9.61 (1H, brs)
9	26.4	3.10 (1H, m)	7-OH		7.54 (1H, brs)

DMSO: Dimethyl Sulfoxide.

**Figure 2:**
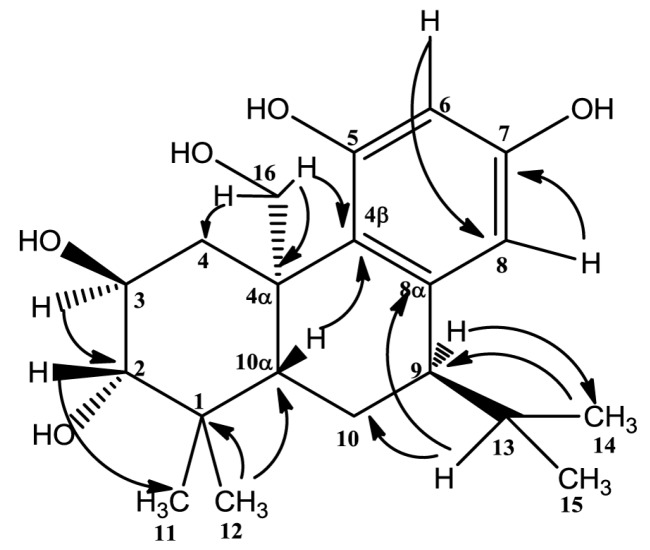
Key HMBC correlations of compound **1** (arrows points from H to C).

**Figure 3:**
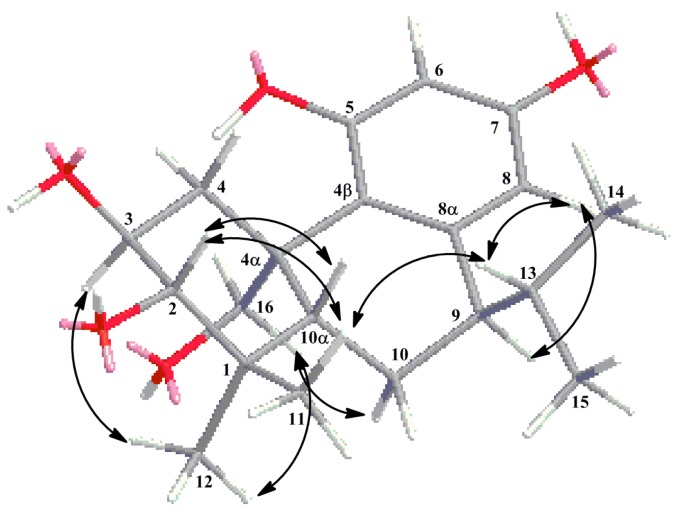
Key ROESY correlations of compound **1**.

^1^H detected heteronuclear multiple bond correlation (HMBC) correlations from H-9 (3.10, 1H, m) to C-14 (22.3), H-13 (2.74, 1H, m) to C-10 (18.1) and C-8α (131.9), H-14 (1.10, 3H, d, *J* = 2.40 Hz) to C-9 (26.4) revealed that the isopropyl in C-9. Key HMBC correlations were given in Figure 2. According to these, the structure **1**, similar to the structure of 2α,3β,11,12-tetrahydroxy-7β,20-epoxy-8,11, 13-abietatriene [10], was established basically. The difference between the two compounds is that the ether bond does not exist in compound **1,** along with the different locations of isopropyl and phenolic hydroxyls. This was the first time to report the isopropyl on the location of C-9. Further correlations from δ_H_ 3.10 (1H, m, H-9) with δ_H_ 3.73 (1H, d, *J* = 9.42 Hz, H-16-I), along with δ_H_ 3.57 (1H, m, H-3) with 4.12 (1H, d, *J* = 9.84 Hz, H-16-II) in the rotating frame overhauser effect spectroscopy (ROESY) spectrum suggested that H-3, H-9, H-16 were alpha-oriented. The correlations from δ_H_ 2.84 (1H, dd, *J* = 3.42, 5.79 Hz, H-2) with δ_H_ 1.30 (1H, s, H-10α), and δ_H_ 2.74 (1H, m, H-13) with δ_H_ 1.30 (1H, s, H-10α) suggested that H-2, H-10α and H-13 were on the same side. C-13 (the carbon that H-13 directly connected) is on the location of C-9 and in the opposite direction of H-9. So the correlations suggested that H-2, H-10α and H-13 were beta-oriented. Key ROESY correlations were given in Figure 3. Based on the above evidences, the structure of compound **1** was established to be 1,2,3,4,4α,9,10,10α-octahydro-(4α-hydroxyymethyl)-1,1-dimethyl-9-(1-methylethyl)-(2*S*,3*S*,4α*R*,9*R*,10α*S*)-2,3,5,7-phenanthrenetertrol and named plebeianiol A.

**Scheme 1:**
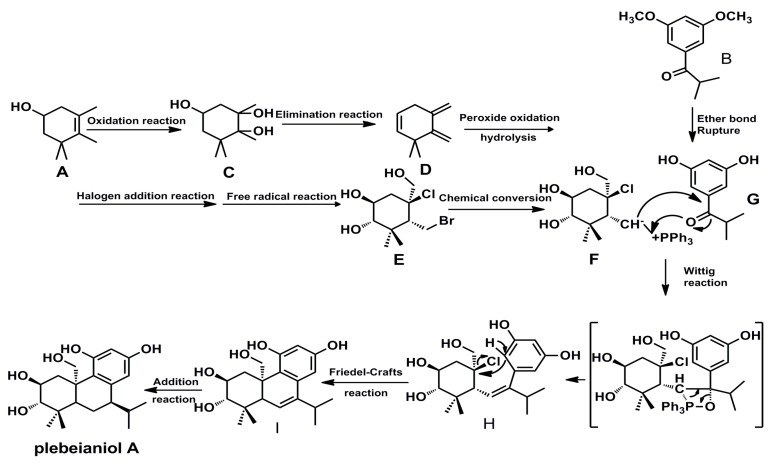
Hypothetical biosynthetic pathway of compound **1**.

In the biosynthetic pathway, monoterpene A (3,4,5,5-tetramethyl-3-cyclohexen-1-ol) and monoterpene derivative B (3′,5′-dimethoxyl-2-methylpropiophenone) may contribute to the formation of compound **1**. The original structure of compound **1** was formed through the combination of F and G. In addition, wittig reaction, friedel-crafts reaction and addition reaction were helpful to the establishment of target compound. The detailed process was showed in Scheme 1.

By comparing the spectral data (MS, ^1^H-NMR, ^13^C-NMR) with the reported values, four known diterpenoids were identified as carnosol (**2**) [11], isocarnosol (**3**) [12], saficinolide (**4**) [13] and 2,11,12-trihydroxy-7,20-epoxy-8,11,13-abietatriene (**5**) [10].

The antioxidant activity of compounds **1**–**5** was evaluated by DPPH radical scavenging activity. The results showed that compounds **1**, **2** and **5** had significant effects with IC_50_ values of 29.6, 28.8 and 20.0 µM, respectively (Table 2). Compared with Vitmain C (Vit. C), these three compounds showed similar antioxidant effects. In order to further evaluate the antioxidant effects, the ability to scavenge ROS in LPS-induced RAW 264.7 macrophages was measured at various concentrations (10, 30 and 100 µM) (Table 3). The results showed that compounds **1**, **2** and **5** had significant effects along with the inhibition rates of 64.8%, 80.3% and 91.3% at the concentration of 100 µM. The results of intracellular ROS assay were similar to DPPH radical scavenging assay. The anti-inflammatory activity tests showed that compounds **1**–**3** inhibited NO production in LPS-induced macrophages with IC_50_ values of 18.0, 21.5 and 23.6 µM, respectively. In addition, RAW 264.7 macrophages were cultured with the obtained compounds at the concentration of 100 µM for 24 h and cell viability was evaluated using MTT assay. The cell viability was in the range of 91.6%–110.5% of the control. These results demonstrated that all compounds did not exhibit substantial cytotoxicity on RAW 264.7 macrophages. The anti-inflammatory experimental data and cell viability are shown in Table 4.

**Table 2:** DPPH radical scavenging activity of isolated compounds.

Compound	5 (µM)	10 (µM)	20 (µM)	30 (µM)	50 (µM)	IC50(µM)
**1**	16.6 ± 3.3	21.0 ± 1.8	39.2 ± 4.1	53.4 ± 8.2	75.4 ± 6.8	29.6
**2**	5.9 ± 1.6	15.4 ± 2.6	29.5 ± 1.9	59.3 ± 4.2	86.1 ± 3.3	28.8
**3**	3.4 ± 2.3	11.5 ± 2.4	24.5 ± 1.2	42.6 ± 6.0	49.0 ± 1.1	>50
**4**	14.5 ± 2.9	15.2 ± 6.0	15.5 ± 1.3	7.0 ± 4.7	13.2 ± 1.5	>50
**5**	9.2 ± 5.8	23.8 ± 6.6	55.4 ± 2.7	72.6 ± 5.7	89.6 ± 1.3	20.0
Vit. C	6.1 ± 4.4	21.6 ± 2.2	43.8 ± 2.6	57.6 ± 5.4	89.1 ± 2.2	26.5

**Table 3:** Effects of isolated compounds on the production of intracellular ROS in LPS-stimulated RAW 264.7 macrophages.

Compound	Inhibition Rate (%)		
	10 (µM)	30 (µM)	100 (µM)
**1**	24.6 ± 2.4 **	56.1 ± 8.2 *	64.8 ± 5.2 **
**2**	29.5 ± 3.1 **	53.2 ± 2.0 **	80.3 ± 1.3 **
**3**	13.5 ± 1.1	16.0 ± 1.9	22.0 ± 1.8 **
**4**	8.1 ± 3.2	16.6 ± 4.4 *	47.1 ± 6.1 **
**5**	13.0 ± 3.3	51.8 ± 2.2 **	91.3 ± 9.2 **
Apocynin	ND	ND	92.9 ± 2.6 **

The results were expressed as the mean ± SD. of three independent experiments. * *p* < 0.05, ** *p* < 0.01, *vs*. model group. ND: Not Determined.

**Table 4:** Effects of compounds on NO production in LPS-stimulated RAW264.7 macrophages and cell viability.

Compound	NO Inhibition Rate (%)				IC_50_	Cell Viability (% *vs.* Control)
	3 (µM )	10 (µM )	30 (µM)	100 (µM)		100(µM)
**1**	18.0 ± 3.2 **	42.9 ± 2.5 **	68.9 ± 5.2 **	86.6 ± 4.9 **	18.0	97.3 ± 4.2
**2**	20.5 ± 2.6 **	32.0 ± 4.8 **	63.3 ± 5.3 **	103.4 ± 5.9 **	21.5	110.5 ± 0.9
**3**	18.8 ± 3.0 **	29.9 ± 5.0 **	59.4 ± 4.7 **	98.1 ± 6.5 **	23.6	106.7 ± 1.5
**4**	6.3 ± 2.4	11.9 ± 5.8	15.8 ± 4.4 *	28.7 ± 5.3 **	>100	101.3 ± 5.0
**5**	5.7 ± 3.8	4.9 ± 7.3	16.4 ± 5.4 *	25.6 ± 4.0 **	>100	91.6 ± 2.3
ibuprofen	ND	ND	32.3 ± 4.2 **	ND	ND	ND

The results were expressed as the mean ± SD. of three independent experiments. * *p* < 0.05, ** *p* < 0.01, *vs*. model group. ND: Not Determined.

## 3. Experimental Section

### 3.1. General Information

IR spectra recorded on a Bruker tensor 27 spectrometer KBr-disks (Bruker, Karlsruhe, Germany). Mass spectra were obtained on a MS 1100 SERIES LC/MSD Trap mass spectrometer (ESI-MS) (Shanghai, China) and HR-ESI-MS were recorded with an Agilent 1260 UPLC DAD 6530 QTOF mass spectrometer (Shanghai, China). Optical rotations were measured with a JASCO P-1020 polarimeter NMR spectra were recorded on Burker ACF-300 NMR spectrometers with TMS as internal standard. Column chromatography was performed on Silica gel H (100–200 mesh, 200–300 mesh, Qingdao Marine Chemical Co., Ltd, Qingdao, China) and Sephadex LH-20 (40–75 µm, Shanghai, China). Thin-layer chromatography was performed on Silica gel GF_254_ (Qingdao Marine Chemical Co., Ltd, Qingdao, China). The absorption was measured using Thermo Scientific Varioskan Flash (Thermo Fisher Scientific Co., Ltd, Shanghai, China). LPS (from *Escherichia coli*) and trypsin were purchased from Sigma-Aldrich (St. Louis, MO, USA). Fetal bovine serum (FBS) was purchased from Hangzhou Sijiqing Biological Engineering Materials Co., Ltd, (Hangzhou, China). The other chemicals used were of analytical grade (Tianjin Chemical Co., Ltd, Tianjin, China).

### 3.2. Plant Material

The air-dried herbs of *S. plebeia* R. Br. were purchased from the Pharmacy of Yi-Feng (Nanjing, China) in July 2013. The herb were collected from Nanning, Guangxi province, China (Production license: 20100109), and identified by Min-Jian Qin, China Pharmaceutical University (Nanjing, China). A voucher specimen (No. 20130702) was deposited in Department of Pharmacology of Chinese Materia Medica, China Pharmaceutical University, Nanjing, China.

### 3.3. Extraction and Isolation

The air-dried herb of *S. plebeia* R. Br. (5.0 kg) was soaked in 80% EtOH (90.0 L) at room temperature for 12 h and extracted under refluxed four times (3 h each). The combined extracts were filtered and solvent was recycled under reduced pressure with a rotary evaporator at 65 °C to obtain a black crude extract (1000.0 g). The extract was suspended in water (3000 mL) and re-extracted by sequentially with petroleum ether (3 × 3000 mL), EtOAc (3 × 3000 mL) and *n*-BuOH (3 × 3000 mL), respectively. Each partitioned fraction was dried at 60 °C under reduced pressure with a rotary evaporator, and the results were 150.0 g (petroleum ether extract), 260.0 g (EtOAc extract) and 200.0 g (*n*-BuOH extract), in turn.

The EtOAc fraction was subjected on the silica gel and the eluent was a gradient system of CH_2_Cl_2_/MeOH (200:1–0:1, *v*/*v*) to give nine fractions (Fr. 1–Fr. 9). Fr. 4 (40.0 g) was rechromatograhed on a silica gel column eluted with CH_2_Cl_2_/MeOH (50:1–0:1, *v*/*v*) to give seven subfractions (Fr. 1_a_–Fr. 1_g_). The subfraction Fr. 1_d_ was rechromatograped on MCI column chromatography to remove the chlorophyll and eluted with MeOH/H_2_O (70:30, *v*/*v*). A yellow-brown solution was obtained and stored under normal temperature. Finally, some yellow crystals were precipitated from the solution. The pure compound **1** (27 mg) was obtained by filtering. The remaining subfractions were rechromatographed on silica gel column eluted with CH_2_Cl_2_/EtOAc (30:1–1:1, *v*/*v*) and purified with Sephadex LH-20 column chromatography (MeOH) to obtain four diterpenoids **2** (200 mg), **3** (45 mg), **4** (13 mg) and **5** (19 mg).

### 3.4. Spectral Data

Compound **1**: C_20_H_30_O_5_, yellow crystal (CHCl_3_-MeOH, 15:1); ${\left[\text{α}\right]}_{\text{D}}^{27}$ +25.8 (*c* 0.1, MeOH); m.p. 232–233 °C; UV (MeOH) λ_max_ (log ε): 211 (3.69) nm; IR (KBr) ν_max_: 3455, 2957, 1641, 1423, 875, 669 cm^−1^; HR-ESI-MS, *m*/*z*: 351.2195 [M + H]^+^; ^1^H-NMR (300 MHz, DMSO-*d*_6_) δ: 9.61 (1H, brs, OH-5), 7.54 (1H, brs, OH-7), 6.53 (1H, brs, H-6), 6.35 (1H, brs, H-8), 4.51 (1H, d, *J* = 3.78 Hz, OH-2), 4.39 (1H, d, *J* = 4.05 Hz, OH-3), 4.12 (1H, d, *J* = 9.84 Hz, H-16-II), 3.73 (1H, d, *J* = 9.42 Hz, H-16-I), 3.57 (1H, m, H-3), 3.36 (1H, d, *J* = 3.39 Hz, H-4-II), 3.10 (1H, m, H-9), 2.84 (1H, dd, *J* = 3.42, 5.79 Hz, H-2), 2.74 (1H, m, H-13), 1.60 (1H, m, H-10-II), 1.50 (1H, m, H-10-I), 1.30 (1H, s, C-10α), 1.11 (3H, d, *J* = 2.49 Hz, H-15), 1.10 (3H, d, *J* = 2.40 Hz, H-14), 1.01 (3H, s), 0.96 (1H, s, H-4-I), 0.78 (3H, s, H-12); ^13^C-NMR (75 MHz, DMSO-*d*_6_) δ: 143.0 (C-5), 141.6 (C-7), 131.9 (C-8α), 129.9 (C-4β), 126.3 (C-7), 117.5 (C-8), 81.9 (C-2), 66.8 (C-3), 65.3 (C-16), 52.1 (C-10α), 43.8 (C-4α), 38.6 (C-1), 38.0 (C-4), 31.7 (C-13), 29.6 (C-11), 26.4 (C-9), 22.4 (C-15), 22.3 (C-14), 18.1 (C-10, 12).

### 3.5. Antioxidant Assay

*DPPH radical assay:* DPPH radical scavenging assay was performed according to previous protocol with modifications [14]. DPPH was dissolved in ethanol at the concentration of 200 µM. One hundred microliters of compounds (5, 10, 20, 30, 50 µM) were added to the 96-well plates, and evaded the light preservation for 30 min after addition 100 µL DPPH. Then the absorbance was measured at 517 nm using Thermo Scientific Varioskan Flash. Vitamin C was used as the positive control. The inhibitory effect was expressed as scavenging rate of alleviation and was calculated as follows:

Scavenging rate (%) = [1 − (A_1_ − A_2_)/A_0_] × 100 (1)

A_1_ refers to the absorbance with various concentrations of test compounds and DPPH, A_2_ refers to the absorbance with various concentrations of test compounds and ethanol, A_0_ refers to the absorbance with distilled water and DPPH.

*Intracellular ROS assay*: Intracellular ROS assay was performed according to the method reported previously [15]. RAW 264.7, a murine macrophage cell line, was obtained from the Pharmacognosy Laboratory of Prof. Mian Zhang, China Pharmaceutical University. Cells were cultured in DMEM containing 10% fetal bovine serum, 100 U/mL penicillin and 100 U/mL streptomycin at 37 °C in a humidified atmosphere containing 5% CO_2_ and 95% air. The cells (1 × 10^5^ cells/well) were pre-incubated in the 96-well plate for 12 h. Then, cells were cultured in normal medium as normal group, or 1 μg/mL LPS as control group, or 1 μg/mL LPS plus different concentration of compounds as test group. After the cells of each group were treated for 6 h, the medium was removed, and the cells were incubated with 50 μL 2,7-Dichlorofluorescein diacetate (DCFH-DA) (10 μM) for 20 min at 37 °C. After that, the cells were washed with PBS carefully three times to remove free DCFH-DA. The absorbance was determined at 525 nm using Thermo Scientific Varioskan Flash. Apocynin was used as the positive control. The inhibitory rate (%) was calculated according to the equation:

Inhibition rate (%) = [1 − (B_1_ − B_0_)/(B_2_ − B_0_)] × 100 (2)

B_1_ refers to the absorbance of control group and B_2_ refers to the absorbance of test group, B_0_ refers to the absorbance of normal group.

### 3.6. Anti-Inflammatory Assay

The production of inflammatory mediators-NO in LPS-stimulated RAW 264.7 macrophages were used to evaluate the anti-inflammatory activity of testing drugs. The nitrite concentration was an indicator of NO production, and measured according to the Griess reaction in the culture medium [16]. After the cells of each group were treated as described above for 24 h, 100 μL of supernatant were mixed with 100 μL of Griess reagents (Reagent A: including 100 µg sulfanilamide and 600 µL 85% H_3_PO_4_ in 10 mL double distilled water; Reagent B: including 10 µg naphthyl ethylenediamine hydrochloride and 10 mL double distilled water; mixed equal volumes of reagents A and B) and standing for 10 min. The absorbance at 540 nm was measured using Thermo Scientific Varioskan Flash. The NO concentrations were calculated by regression analysis of a standard curve with sodium nitrite as a standard. The ibuprofen as the positive medicine was measured at the concentration of 30 µM. Inhibitory rate (%) was calculated according to the formula:

Inhibition rate (%) = [1 − (C_1_ − C_0_)/(C_2_ − C_0_)] × 100 (3)

C_1_ refers to the absorbance of control group and C_2_ refers to the absorbance of test group, C_0_ refers to the absorbance of normal group.

### 3.7. Cell Viability Assay

The MTT assay was used to evaluate the cell viability, as previously reported [17]. RAW 264.7 macrophages were seeded at a density of 1 × 10^5^ cells /mL in 96-well plates and cultured for 24 h with the test compound (100 µM) at 37 °C in a 5% CO_2_ incubator. Subsequently, 20 μL of MTT solution (5 mg/mL) was added to each well and incubated for 4 h at 37 °C and the resulted crystals were dissolved in DMSO. The optical density was measured at 490 nm using Thermo Scientific Varioskan Flash.

## 4. Conclusions

In conclusion, five diterpenoids, including a new skeleton plebeianiol A, carnosol, isocarnosol, saficinolide and 2,11,12-trihydroxy-7,20-epoxy-8,11,13-abietatriene were identified from the EtOAc extract of *S. plebeia* R. Br. Compounds **1**, **2** and **5** showed significant antioxidant activities. In addition, compounds **1**–**3** showed significant anti-inflammatory activities. These results showed that compounds **1**, **2** had significant effects on antioxidant and anti-inflammatory activities.

## Supplementary Materials

Supplementary materials can be accessed at: http://www.mdpi.com/1420-3049/20/08/14879/s1.
