# Supplementary material for: Diterpenoids from Saliva plebeia R. Br. and Their Antioxidant and Anti-Inflammatory Activities
Source: Molecules. 2015 Aug 14;20(8):14879–88. doi: 10.3390/molecules200814879 (PMC6332173; doi:10.3390/molecules200814879)
Supplement: Supplementary file 1 [file molecules-20-14879-s001.pdf]

# Supplementary Material

# Diterpenoids from *Salvia plebeia* R. Br. and Their Antioxidant and Anti-Inflammatory Activities

### NMR Spectra of Compound 1

Herein, we provide the original UV spectra, IR spectra, 1D and 2D spectra and NMR chemical shifts of Compound **1**.

The HR-ESI-MS spectrum see Figure S1, UV spectrum see Figure S2, IR spectrum see Figure S3, $^{1}$H-NMR spectrum see Figures S4, $^{13}$C-NMR see Figure S5, HSQC spectrum see Figure S6, HMBC spectrum see Figure S7, and ROESY see Figure S8.

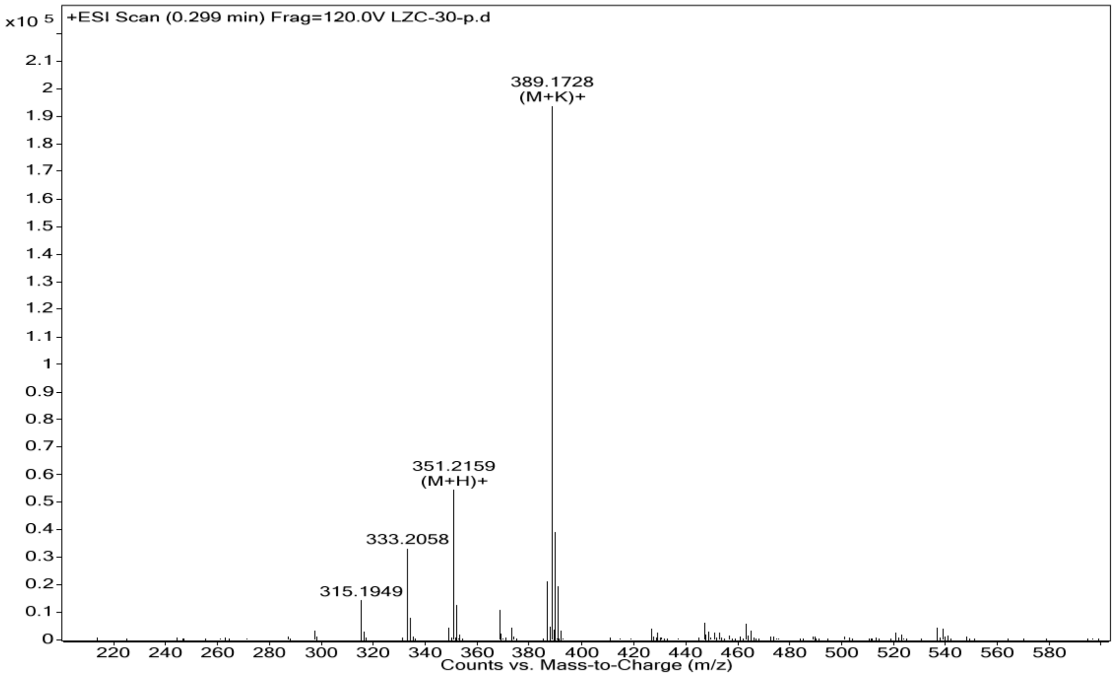


MS Formula Results: + Scan (0.299 min) (LZC-30-p.d)

| m/z | Ion | Formula | Abundance |
|---|---|---|---|
| 351.2159 | (M+H)+ | C20 H31 O5 | 54501 |

| Best | Formula (M) | Ion Formula | Calc m/z | Score | Cross Score | Mass | Calc Mass | Diff (ppm) | Abs Diff (ppm) | Abund Match | Spacing Match | Mass Match | m/z | DBE |
|---|---|---|---|---|---|---|---|---|---|---|---|---|---|---|
| ☑ | C20 H30 O5 | C20 H31 O5 | 351.2166 | 97.92 | | 350.2086 | 350.2093 | 2.05 | 2.05 | 99.35 | 98.49 | 96.78 | 351.2159 | 6 |
| ☐ | C18 H28 N3 O4 | C18 H29 N3 O4 | 351.2153 | 97.54 | | 350.2086 | 350.208 | -1.8 | 1.8 | 97.12 | 98.1 | 97.51 | 351.2159 | 6.5 |
| ☐ | C19 H24 N7 | C19 H25 N7 | 351.2166 | 97.36 | | 350.2086 | 350.2093 | 2.01 | 2.01 | 98.79 | 96.57 | 96.9 | 351.2159 | 11.5 |
| ☐ | C21 H26 N4 O | C21 H27 N4 O | 351.2179 | 87.81 | | 350.2086 | 350.2107 | 5.85 | 5.85 | 97.74 | 98.39 | 76.56 | 351.2159 | 11 |
| ☐ | C16 H26 N6 O3 | C16 H27 N6 O3 | 351.2139 | 86.61 | | 350.2086 | 350.2066 | -5.64 | 5.64 | 92.94 | 96.2 | 78.02 | 351.2159 | 7 |
| ☐ | C23 H28 N O2 | C23 H29 N O2 | 351.2193 | 73.59 | | 350.2086 | 350.212 | 9.7 | 9.7 | 95.01 | 98.99 | 48.05 | 351.2159 | 10.5 |
| ☐ | C14 H24 N9 O2 | C14 H25 N9 O2 | 351.2126 | 70.42 | | 350.2086 | 350.2053 | -9.49 | 9.49 | 86.94 | 92.32 | 49.55 | 351.2159 | 7.5 |
| ☐ | C15 H30 N2 O7 | C15 H31 N2 O7 | 351.2126 | 69.7 | | 350.2086 | 350.2053 | -9.45 | 9.45 | 79.4 | 97.7 | 49.88 | 351.2159 | 2 |

| m/z | Ion | Formula | Abundance |
|---|---|---|---|
| 389.1728 | (M+K)+ | C20 H30 K O5 | 193627.7 |

| Best | Formula (M) | Ion Formula | Calc m/z | Score | Cross Score | Mass | Calc Mass | Diff (ppm) | Abs Diff (ppm) | Abund Match | Spacing Match | Mass Match | m/z | DBE |
|---|---|---|---|---|---|---|---|---|---|---|---|---|---|---|
| ☑ | C20 H30 O5 | C20 H30 K O5 | 389.1725 | 98.59 | | 350.2096 | 350.2093 | -0.75 | 0.75 | 97.81 | 97.5 | 99.6 | 389.1728 | 6 |
| ☐ | C19 H24 N7 | C19 H24 K N7 | 389.1725 | 97.84 | | 350.2096 | 350.2093 | -0.78 | 0.78 | 93.5 | 99.57 | 99.57 | 389.1728 | 11.5 |
| ☐ | C21 H26 N4 O | C21 H26 K N4 O | 389.1738 | 93.79 | | 350.2096 | 350.2107 | 3.06 | 3.06 | 89.31 | 99.44 | 93.64 | 389.1728 | 11 |
| ☐ | C18 H28 N3 O4 | C18 H28 K N3 O4 | 389.1711 | 93.13 | | 350.2096 | 350.208 | -4.59 | 4.59 | 99.61 | 99.14 | 86.23 | 389.1728 | 6.5 |
| ☐ | C23 H28 N O2 | C23 H28 K N O2 | 389.1752 | 81.46 | | 350.2096 | 350.212 | 6.9 | 6.9 | 84.18 | 97.94 | 71.58 | 389.1728 | 10.5 |
| ☐ | C16 H26 N6 O3 | C16 H26 K N6 O3 | 389.1698 | 81.04 | | 350.2096 | 350.2066 | -8.44 | 8.44 | 99.79 | 99.29 | 60.66 | 389.1728 | 7 |

**Figure S1.** HR-ESI-MS spectrum of compound **1**.

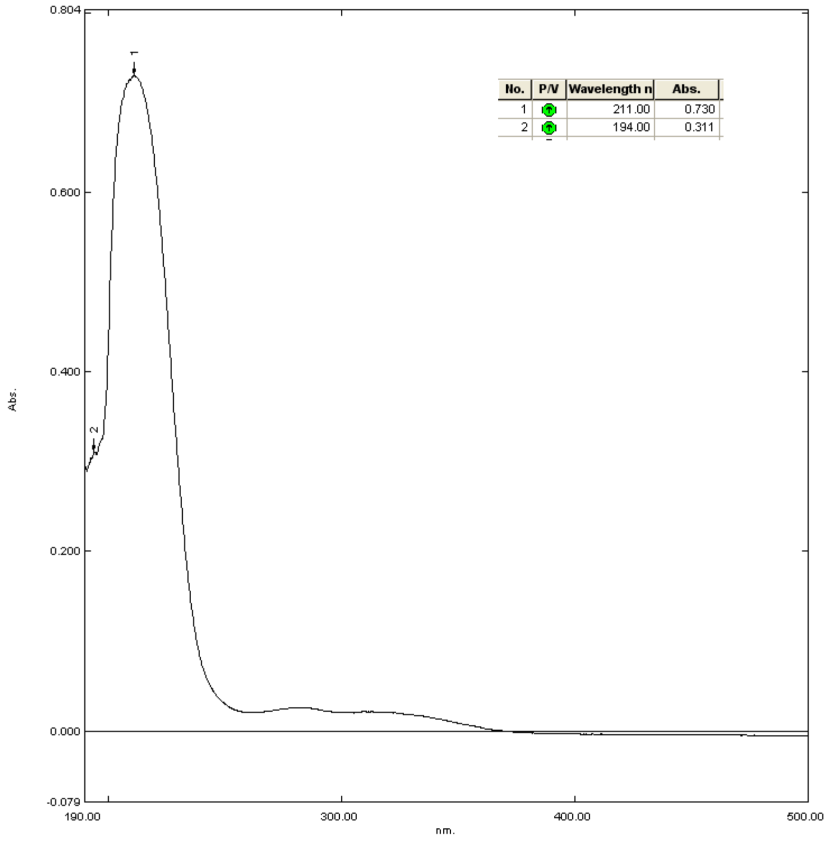


**Figure S2.** UV spectrum of compound **1**.

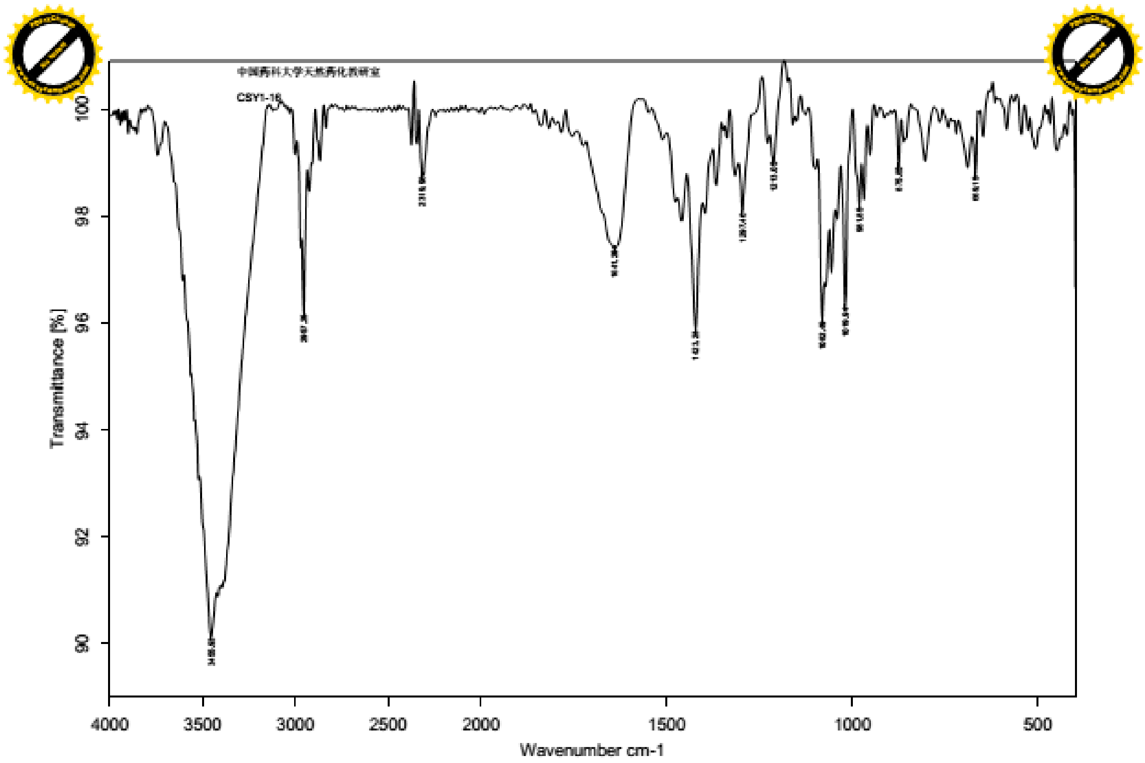


**Figure S3.** IR spectrum of compound **1**.

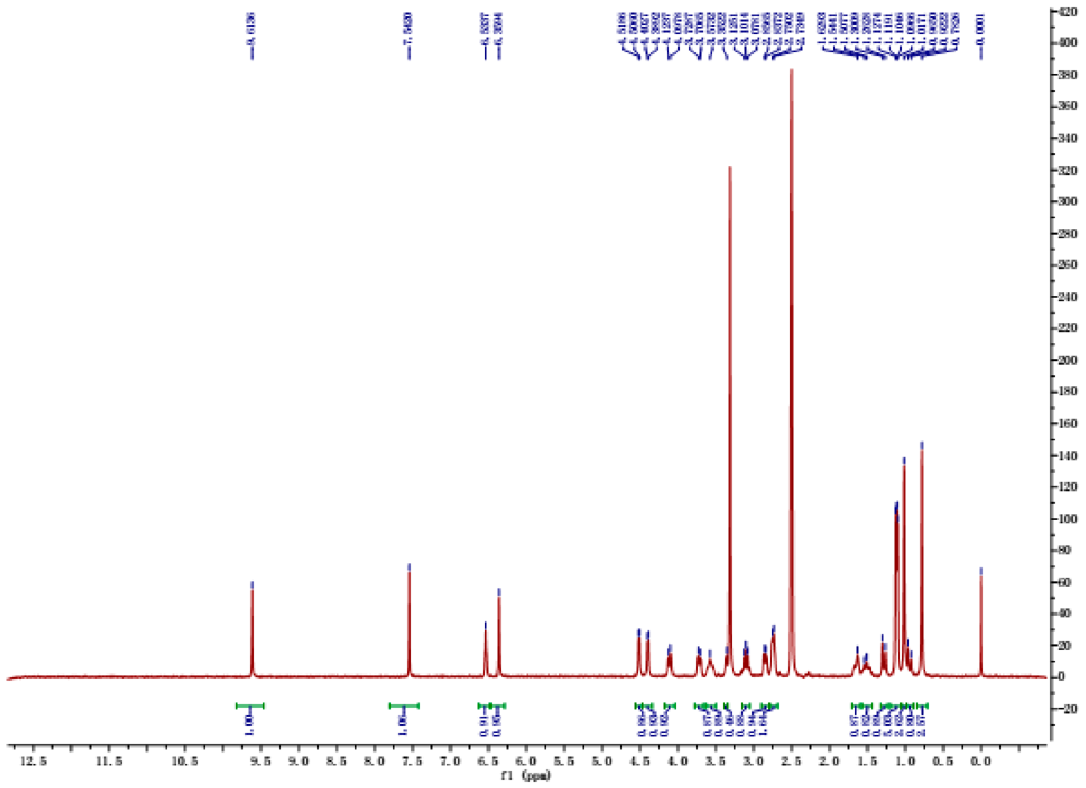

**Figure S4.** $^1$H-NMR spectrum of compound **1** (DMSO-$d_6$, 300 MHz).

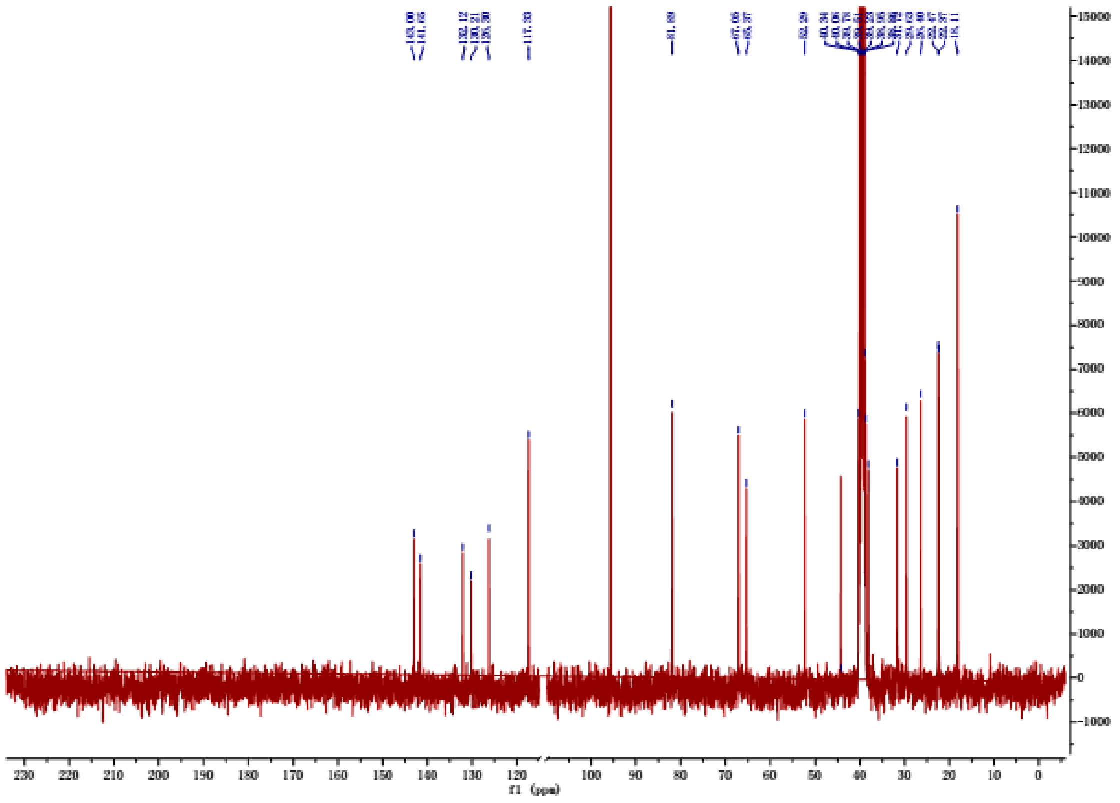

**Figure S5.** $^{13}$C-NMR spectrum of compound **1** (DMSO-$d_6$, 75 MHz).

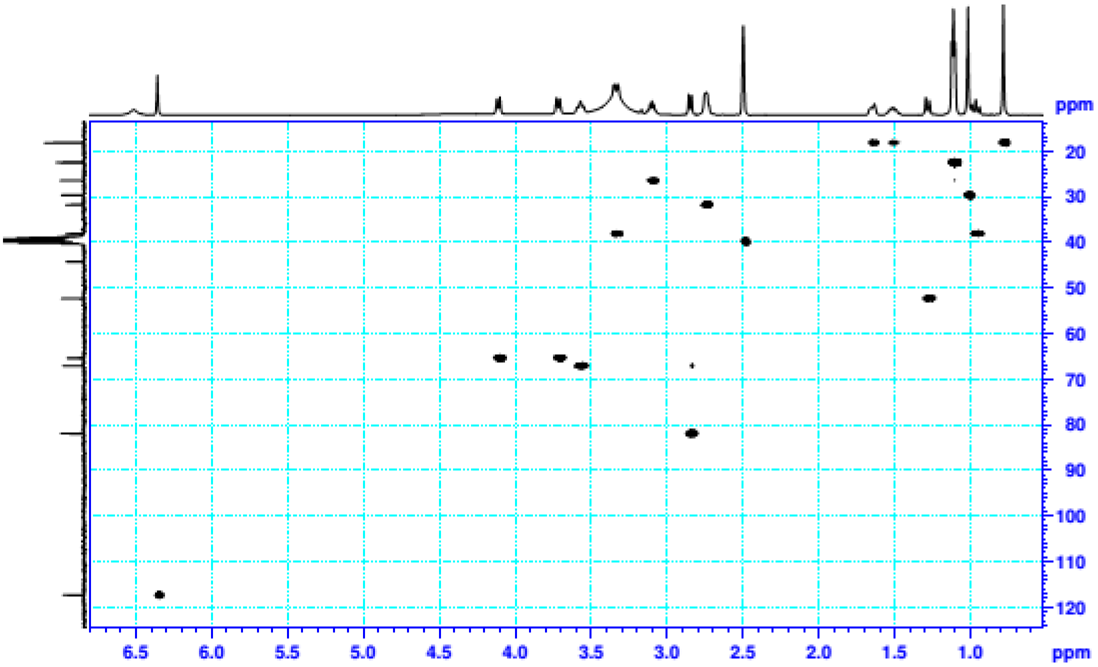


**Figure S6.** HSQC correlations of compound **1** (DMSO-$d_6$, 500 MHz).

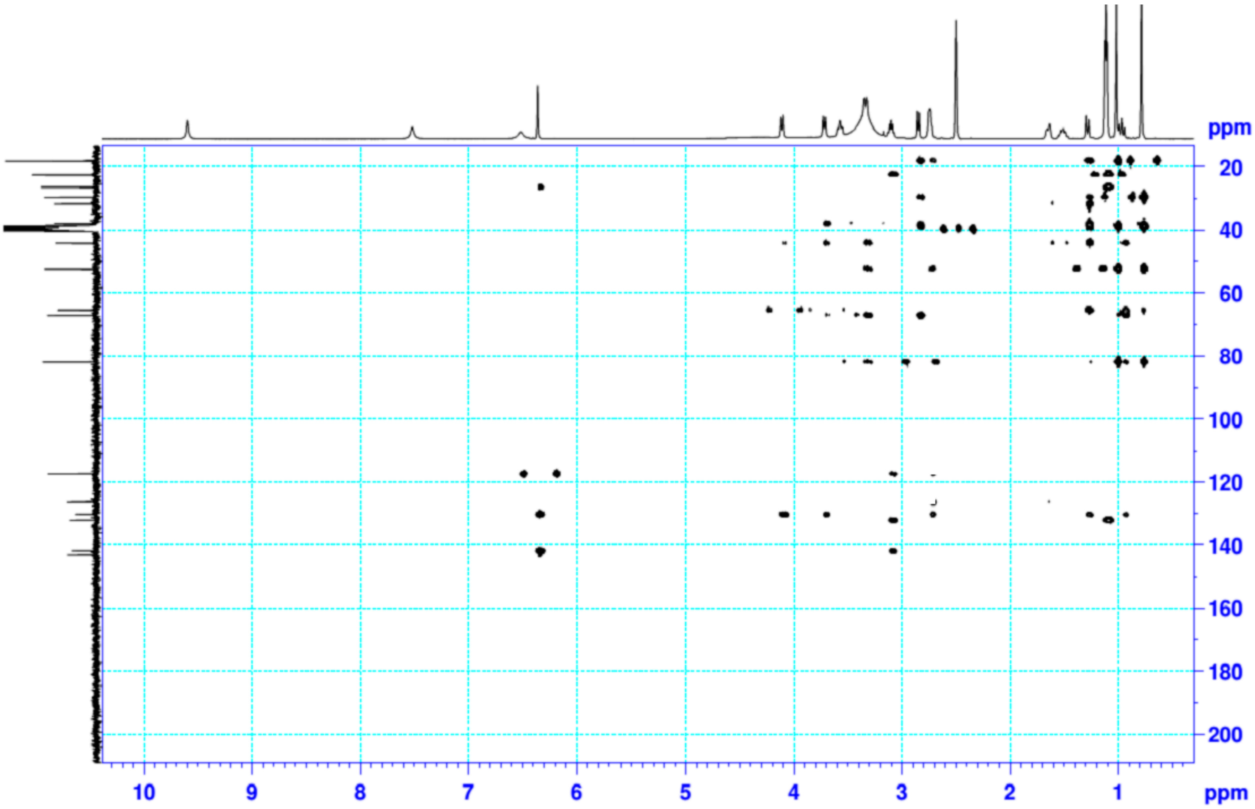


**Figure S7.** HMBC correlations of compound **1** (DMSO-$d_6$, 500 MHz).

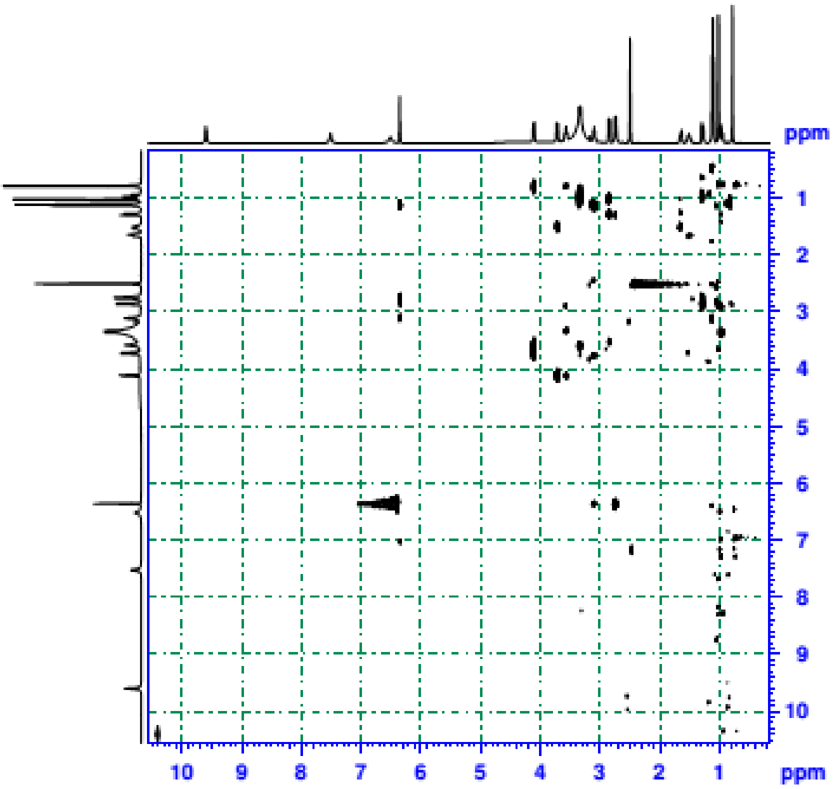


**Figure S8.** ROESY correlations of compound **1** (DMSO-$d_6$, 500 MHz).
